# Efficacy of Bi Qi Capsules in combination with methotrexate in the treatment of rheumatoid arthritis: A protocol of double-blind randomized controlled trial

**DOI:** 10.1097/MD.0000000000025790

**Published:** 2021-05-21

**Authors:** Weizhong Ding, Shirong Chen, Xuexiang Shi, Yang Zhao

**Affiliations:** Department of Orthopedic Surgery, The Second Affiliated Hospital, Chongqing Medical University, Chongqing, China.

**Keywords:** Bi Qi capsule, clinical randomized controlled trials, methotrexate, protocol, rheumatoid arthritis

## Abstract

**Background:**

It is known that Bi Qi Capsules (BQC) have synergistic effects when combined with Methotrexate, but there is a lack of clinical studies on the long-term efficacy and safety of the combination of the 2 in the treatment of rheumatoid arthritis (RA). Therefore, the purpose of this randomized controlled trial was to evaluate the long-term efficacy and safety of this treatment.

**Methods:**

This was a prospective, double-blind, single-simulation, randomized controlled trial investigating the efficacy and safety of BQC in combination with Methotrexate in the treatment of RA, and was approved by the Clinical Research Ethics Committee of the hospital. Patients were randomized in a 1:1 ratio to either the observation or control group and were respectively followed up for 6 months after receiving 12 weeks of treatment. The observation indexes included: total effective rate, DAS-28 score, inflammatory indexes, and adverse reactions. Finally, the collected data was statistically analyzed by SPSS version 18.0.

**Discussion:**

This study evaluated the long-term efficacy of BQC in combination with Methotrexate in the treatment of RA. The trial results of this study will provide new ideas for choosing a combination of Chinese and Western medicine protocols for the treatment of RA.

**Trial registration:**

OSF Registration number: DOI 10.17605/OSF.IO/U85GX.

## 1 Introduction

Rheumatoid arthritis is a chronic autoimmune joint disease characterized by inflammatory cell infiltration and synovial proliferation, accompanied by injury to articular cartilage and subchondral bone, ultimately leading to joint dysfunction and even deformity.^[1,2]^ As a persistent disease, RA not only leads to a lower quality of life, but also imposes a heavy burden on society and the economy,^[3]^ with a worldwide prevalence of about 0.3% to 1%,^[4]^ associated with a variety of chronic diseases and huge costs of care.^[5]^

There is no treatment that could utterly cure RA, but the disease is mainly improved by non-steroidal anti-inflammatory drugs, conventional disease-modifying antirheumatic drugs, glucocorticoids, and biological DMARDs to control and delay the progression of the disease and improve the quality of lives of patients.^[6]^ The American College of Rheumatology recommends that antirheumatic drugs (DMARDs) could be used for the treatment of RA.^[7]^ Methotrexate (MTX) is one of the most widely used DMARDs and the main drug for the treatment of RA.^[8]^ However, the use of a single MTX in many patients is not effective in controlling the activity of RA and relieving clinical symptoms.^[9]^ Therefore, how to optimize RA treatment strategies concerns many clinicians.

Traditional Chinese Medicine offers a variety of inexpensive and effective methods for the treatment of RA, and is often used as a complementary therapy in China.^[10]^ BQC is a commonly used Chinese materia medica preparation for the treatment of RA in China and has been approved by the State Food and Drug Administration as a complementary therapy for RA due to its good efficacy and low side effects.^[11]^ BQC consists of Radix Notoginseng (San Qi), Strychni Semen (Ma Qian Zi), Codonopsis pilosula (Franch.) Nannf (Dang Shen), Rhizoma Atractylodis Macrocephalae (Bai Zhu), Radix Salviae Miltiorrhizae (Dan Shen), Rhizoma Chuanxiong (Chuan Xiong), and Radix Glycyrrhiza (Gan Cao), which have significant anti-inflammatory, antiswelling and analgesic effects.^[12]^ Modern pharmacological studies have shown that BQC have immunosuppressive effects, reducing abnormally elevated immunoglobulin levels, promoting the conversion of rheumatoid factor to negative, and also inhibiting the proliferation of vascular endothelial cells and smooth muscle cells.^[13]^ Some studies have found that the combination of BCQ with MTX for the treatment of RA improves the efficacy and promotes the improvement of clinical symptoms and inflammatory factor levels without increasing adverse effects.^[11]^ However, a direct comparison of BQC combined with MTX vs MTX alone is lacking, and the short follow-up period fails to support the evaluation of the long-term efficacy of this combination regimen. Therefore, we proposed to evaluate the long-term efficacy and safety of BQC in combination with MTX in the treatment of RA in this randomized controlled trial.

## 2 Materials and methods

### 2.1 Study design

**Figure 1:**
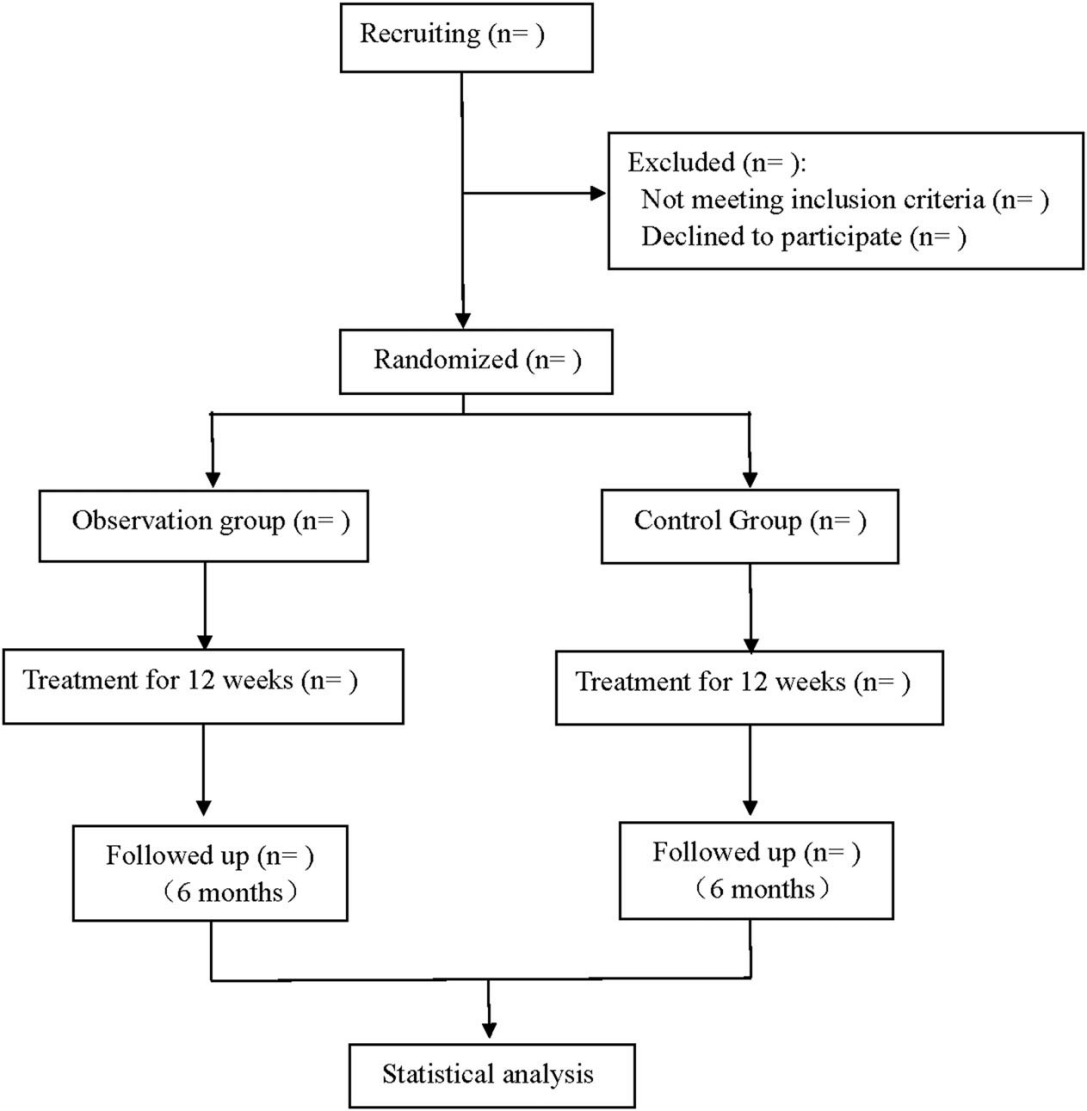
Flow diagram.

This was a prospective, double-blind, single-simulation, randomized controlled trial to investigate the long-term efficacy and safety of BQC in combination with MTX in the treatment of RA, and this study followed the comprehensive trial reporting criteria,^[14]^ the flow chart of which was shown in Figure 1.

### 2.2 Ethics and registration

The study protocol was in accordance with the Declaration of Helsinki and reviewed by Clinical Research Ethics Committee. This experiment has been registered with (OSF) (registration number: DOI 10.17605/OSF.IO/U85GX). Prior to randomization, all patients were required to sign an informed consent form, and they could choose at any time whether to continue or not.

### 2.3 Sample size

The sample size calculation for this study was based on the scores of the total effective rate^[15]^ after 8 weeks of treatment. According to the results of the pretest, the total effective rate of the experimental group was 0.63 and the total effective rate of the control group was 0.41. Set α = 0.025, one-sided test, β = 0.10. The PASS15.0 software calculated that 85 participants were needed in each group, and the estimated dropout rate was 10%, and 95 participants would be included in each group.

### 2.4 Patients

#### 2.4.1 Inclusion criteria

1. Patients age 18 to 65 years old;
2. The diagnostic criteria for RA are met (refer to the 2010 American College of Rheumatology and European League Against Rheumatism criteria for RA^[16]^);
3. Imaging results suggested Class I, II or III disease (according to the 1987 American Rheumatism Association classification standard^[17]^);
4. Patients who have not taken relevant drugs and undergone other treatments within the last 1 month;
5. Patients who were compliant and signed the informed consent form.

#### 2.4.2 Exclusion criteria

1. With important organ damage or malignant tumors, infectious diseases, hematologic diseases;
2. With other rheumatic diseases;
3. Women who are pregnant or breastfeeding;
4. Comorbid active gastrointestinal disease or diagnosis of esophageal or peptic ulcer within the last month;
5. Hypersensitivity to the drugs used in this study.

### 2.5 Study design

Eligible participants were randomly assigned to the treatment or control group in a 1:1 ratio using a central network-based randomization tool. Random sequences were generated by statisticians who did not involve in trial implementation or statistical analysis using SAS 9.3 software (SAS Institute, Cary, NC). Clinical research coordinators entered participant information on a tablet computer and were given a random number. The research assistant obtained the participant's assignment from the computer. Throughout the study, research assistants were responsible for screening, recruiting participants, and assigning random numbers to participants who had been enrolled. Participants, researchers, research assistants, and statistical analysts were unaware of the allocation throughout the process.

### 2.6 Interventions

1. Observation group: Given BQC (Tianjin Daren Tang Jingwanhong Pharmaceutical Co., Ltd., China, GMP Z10910026, 0. 3 g/capsule) 4 capsules per time, 2 times per day, taken orally; Given MTX Tablets (Shanghai Xinyi Pharmaceutical Co., Ltd., China, GMP H31020644, 2.5 mg/tablet) 10 mg per dose, 1 time per week, orally. Continue for 12 weeks.
2. Control group: MTX Tablets (Shanghai Xinyi Pharmaceutical Co., Ltd., China, GMP H31020644, 2.5 mg/tablet), 10 mg per dose, 1 time per week, orally. BQC Mimetic, (consisting mainly of starch, lactose and dextrin), which had the same shape, size and color as BQC, was prepared and supplied by Jiangsu Famaxon Medical Technology Co., Ltd. in China. 4 capsules per dose, 2 times per day, orally. Duration: 12 weeks.

### 2.7 Outcomes and measurements

#### 2.7.1 Primary outcome indicators

Total effective rate: according to the American College of Rheumatology definition of improvement in RA^[15]^: Ineffective as improvement in clinical symptoms, C-reactive protein, sedimentation and rheumatoid factor <20%; Effective as 20% to 70% improvement in symptoms, C-reactive protein, sedimentation and rheumatoid factor; Significant effect was defined as >70% improvement in symptoms, C-reactive protein, blood sedimentation and rheumatoid factor. Total effective rate = (number of effective cases + number of effective cases)/total number of cases × 100%.

#### 2.7.2 Secondary outcome indicators

1. DAS-28 score^[18]^: a DAS-28 score > 5.1 indicating high disease activity; 3.2< DAS-28 score≤5.1 indicates moderate disease activity; 2.6< DAS-28 score≤3.2 indicates low disease activity; DAS-28 score≤2.6 indicates basic disease remission.
2. Inflammatory factor levels: this includes serum interleukin 17, tumor necrosis factor, and rheumatoid factor.
3. Adverse reactions: including nausea and vomiting, dizziness, abdominal pain, etc.

All the above observations were recorded at baseline, week 4, 8, and 12 by specially trained assessors. All patients were followed up for 6 months and the same assessments were completed in the outpatient clinic at 1-month intervals after the end of treatment.

### 2.8 Data collection and management

One or 2 assistants conducted the full data collection and recording. Personal information about potential and enrolled participants was collected, shared and stored in a separate repository to protect confidentiality before, during and after the trial. The access to the database was restricted to the researchers in this study team.

### 2.9 Statistical analysis

The collected data was statistically analyzed by SPSS 18.0 software. The Chi-Squared test was used for the count data; The mean ± standard deviation ($\bar{x}$ *±* *S*) was used for the measurement data; The independent sample *t* test was used for the normal distribution; The Mann-Whitney *U* test was used for the skewed distribution; The differences were considered statistically significant at *P* < .05.

## 3 Discussion

RA is the most common inflammatory arthritis, a chronic autoimmune disease characterized by symmetric, persistent synovitis, and destructive polyarthritis,^[19]^ often accompanied by extra-articular organ involvement and positive serum rheumatoid factor, which can lead to joint deformity and loss of function in severe cases, and RA is characterized by complex etiology and recurrent course.^[20,21]^ Therefore, effective treatment is a serious clinical problem that needs to be addressed urgently.

Traditional Chinese medicine has been practiced in China for thousands of years and it is playing an important role in complementary and alternative therapies.^[22]^ Clinical studies have shown that BQC can inhibit the expression of interleukin (IL)-4, interferon-γ, IL-1 and other inflammatory cytokines,^[23]^ improve the clinical symptoms of patients with rheumatoid arthritis, and also significantly reduce synovial hyperplasia), pannus formation, and destruction of articular cartilage and bone.^[6]^ Animal studies have found that BQC exerts a protective effect on articular cartilage by downregulating cartilage oligomeric matrix protein levels in serum, cartilage oligomeric matrix protein expression in synovium and cartilage, and inhibiting inflammatory infiltration, synovial proliferation, and joint destruction.^[24]^ MTX is a commonly used DMARDs class of drugs in clinical practice, and clinical studies have demonstrated synergistic effects of MTX in combination with paralysis capsules,^[11,13]^ but rigorous randomized, double-blind controlled studies to evaluate their long-term efficacy are lacking. Therefore, a prospective, randomized, double-blind, single-simulation randomized controlled study would be conducted to investigate the long-term efficacy and safety of BQC in combination with MTX compared to MTX alone in the treatment of RA. More multicenter, large-sample studies on this topic were also needed because this study was a singlecenter, randomized controlled study with regionalization of the included population, which may have an impact on the results.

## Author contributions

**Conceptualization:** Weizhong Ding and Xuexiang Shi.

**Data curation:** Weizhong Ding and Shirong Chen.

**Formal analysis:** Xuexiang Shi.

**Funding acquisition:** Shirong Chen.

**Software:** Shirong Chen and Yang Zhao.

**Investigation:** Xuexiang Shi.

**Resources:** Xuexiang Shi, Yang Zhao.

**Supervision:** Xuexiang Shi and Yang Zhao.

**Writing – original draft:** Weizhong Ding and Shirong Chen.

**Writing – review & editing:** Weizhong Ding and Shirong Chen.
